# Antimicrobial Evaluation of Chlorophyll-Containing Nettle Extract Both in Free Form and Incorporated into Poly-3-Hydroxybutyrate

**DOI:** 10.3390/polym17182507

**Published:** 2025-09-17

**Authors:** Polina M. Tyubaeva, Ivetta A. Varyan, Sergei I. Obydennyi, Vasily A. Merzlikin, Svetlana G. Karpova, Olga A. Gruznova, Dmitry V. Gruznov, Ekaterina N. Shuteeva, Nikolay N. Kuvshinchikov, Nikolay I. Popov, Anton V. Lobanov, Ivan A. Abramov, Andrey P. Sergeev, Anzhelika V. Zagaynova, Anatoly A. Olkhov

**Affiliations:** 1 Department of Physical Chemistry of Synthetic and Natural Polymer Compositions, Emanuel Institute of Biochemical Physics, Russian Academy of Sciences, 4 Kosygina St., 119334 Moscow, Russia; vasiliy.merzl@bk.ru (V.A.M.); karpova@sky.chph.ras.ru (S.G.K.); aolkhov72@yandex.ru (A.A.O.); 2 Academic Department of Innovational Materials and Technologies Chemistry, Plekhanov Russian University of Economics, 36 Stremyanny Per., 117997 Moscow, Russia; 3 Dmitry Rogachev National Medical Research Center of Pediatric Hematology, Oncology and Immunology of Ministry of Healthcare of the Russian Federation, 117198 Moscow, Russia; obydennyj@physics.msu.ru; 4 Centre for Theoretical Problems of Physicochemical Pharmacology, 119334 Moscow, Russia; 5 Laboratory of Liquid-Phase Oxidation, N.N. Semenov Federal Research Center for Chemical Physics, Russian Academy of Sciences, 4 Kosygina Street, 119334 Moscow, Russia; gruznova_olga@bk.ru; 6 Laboratory of Veterinary Sanitation, All-Russian Research Institute of Veterinary Sanitation, Hygiene and Ecology—Branch of Federal State Budget Scientific Institution “Federal Scientific Center—K.I. Skryabin, Ya.R. Kovalenko All-Russian Research Institute of Experimental Veterinary Medicine, Russian Academy of Sciences”, 5 Zvenigorodskoye Highway, 123022 Moscow, Russia; 79164422245@yandex.ru (D.V.G.); ekashyt@mail.ru (E.N.S.); kuvshinchikov1@yandex.ru (N.N.K.); dezlab@mail.ru (N.I.P.); 7 Department of General Chemistry, Moscow Pedagogical State University, 1/1 Malaya Pirogovskaya Street, 119435 Moscow, Russia; av.lobanov@mpgu.su; 8 Laboratory of Microbiology and Parasitology, Centre for Strategic Planning and Management of Biomedical Health Risks of Federal Medical Biological Agency, Pogodinskaya Str., 10/1, 119121 Moscow, Russia; iaabramov@cspfmba.ru (I.A.A.); azagaynova@cspfmba.ru (A.V.Z.); 9 Department of Systems Biology and Bioinformatics, Centre for Strategic Planning and Management of Biomedical Health Risks of Federal Medical Biological Agency, Pogodinskaya Str., 10/1, 119121 Moscow, Russia; asergeev@cspfmba.ru

**Keywords:** poly-3-hydroxybutyrate, chlorophyll, electrospinning, antibacterial properties, supramolecular structure

## Abstract

This work is devoted to the electrospinning of biocompatible fibrous matrixes for microbial wound therapy. The problem of treating staphylococcal-infected wounds remains urgent. In this study, we propose a new approach to the use of the chlorophyll (Chl) and poly-3-hydroxybutyrate (PHB) composite system in the treatment of infected wounds. The structure and properties of the electrospun polymer matrix based on PHB modified with various concentrations of Chl was investigated by SEM, confocal microscopy, DSC, EPR. The release rate, fluorescence, and antimicrobial activity of Chl incorporated into PHB were studied. The high efficiency of the developed materials was shown with the participation of laboratory animals.

## 1. Introduction

The microorganisms’ adaptability to the effects of antibiotics is a major challenge for the pharmaceutical and biotechnology industries worldwide. Moreover, an inappropriate and an irrational use of antimicrobial drugs leads to both a reduction in the therapeutic effect and serious economic costs [1,2,3].

In this regard, the search for and development of alternative approaches is actively being carried out. For instance, antibacterial materials based on hydrogels [4,5], including alginate hydrogels modified with imidazolium-based bi-ionic liquids and silver nanoparticle [6], core–shell metal nanoparticles [7], photocatalytic and photodynamic agents [8,9,10,11,12] have already demonstrated high potential. Medicinal plants and their extracts that are biocompatible with the human and animal organisms and non-toxic also seem promising [13].

Various parts of the plant contain substances responsible for antitumor, antioxidant and antimicrobial effects [14,15,16]. There is evidence that pigments, including chlorophylls (Chl), have the properties mentioned. For example, A. Ahmadi et al. noted the inhibitory effect of a mixture of Chl*a* and Chl*b* extracted from alfalfa on *L. monocytogenes*, *S. aureus* and *S. typhi*. The possibility of replacing antibiotics with chlorophyll extract has been suggested [17]. L.E. Maekawa et al. demonstrated the fungicidal activity of chlorophyll extract in the treatment of oral candidiasis [18]. The antimicrobial potential of Chl extracted from the algae *Sargassum polycystum* was confirmed in experiments with antibiotic-resistant strains of *S. aureus* and *E. coli* [19]. E. Arslan et al. also reported the antibacterial activity of Chl obtained from green microalgae against *P. aeruginosa*, *S. aureus* and *E. coli* [20]. The antitumor, anti-inflammatory and antioxidant activity of chlorophyll is described in the works of D.C. Vesenick et al., U.M. Lanfer-Marquez et al., V. da Silva Ferreira et al. [21,22,23].

Chl is found in the chloroplasts of all photosynthetic organisms and is responsible for the green color. Currently, the following types of Chl are known: *a*, *b*, *c1*, *c2*, *d*, and *f* [24,25]. The plants mainly contain Chl*a* and Chl*b* in an approximate mass ratio of 3:1 [26]. The structure of both Chls differs in the radical: the -CH_3_ group is characteristic of Chl*a*, and -CHO—of Chl*b* [17].

The production of plant extracts, including Chl-containing ones, should be profit-making. Hence, suitable raw materials are widely distributed crops, the cultivation of which does not require large time and material costs [27]. A. Ahmadi et al. reported the feasibility of using alfalfa (*Medicago sativa* L.) for these purposes [17]. The preparation of aqueous extracts from 64 species of weeds (*Chenopodium murale*, *Falcaria vulgaris*, *Ranunculus asialieus*, *Sisymbrium irio*, etc.) is described in the work [28]. L.E. Maekawa et al. used various seaweeds (*Gracilaria corticata*, *Turbinaria ornata*, *Sargassum polyphyllum*, *Padina tetrastomatica*, *Gelidiopsis* sp.) [29]. The stinging nettle (*Urtica dioica*) is also a promising raw material for extraction. It is a perennial herbaceous plant that grows independently in the temperate zone of both hemispheres of the Earth [30]. It has been known as an effective remedy for arthritis, rheumatism, gout, anemia, and eczema since ancient times [31,32,33,34,35]. Nowadays, a nettle is utilized in the powders, essential oils and extracts forms [35,36,37,38]. To enhance the beneficial properties of nettle, there are several approaches to including it in various carriers. For example, to treat infected skin ulcers, a dressing in the nanocomposite sponge’s form based on sodium alginate and polyvinyl alcohol with nettle as an antibacterial and hemostatic agent was developed [39]. H.-R. Alizadeh-Otaghvar’s study noted the beneficial effect of nettle in the silk fibroin composition on wound healing [40]. S.M.T. Gharibzahedi and S. Mohammadnabi reported high antibacterial activity of nettle essential oil nanoemulsions against Gram-positive bacteria [41]. According to E. Atiek et al., an aqueous extract of nettle leaves in the composition of TiO_2_/ZnO nanoparticles demonstrated a significant inhibitory effect on *S. aureus*, *S. pyogenes*, *P. aeruginosa*, and *E. coli*. M. Flórez et al. enriched chitosan films with an aqueous extract of nettle to obtain an antioxidant agent [42]. In addition, a nettle is commercial source of a Chl [26,34]. According to some data, the Chl content in its leaves is up to 5 g/kg [26,43].

Consequently, a particularly auspicious and pertinent trajectory entails the integration of Chl into polymeric carriers, meticulously engineered to facilitate regulated release [44]. To this end, polymers that degrade quickly in contact with a living environment, such as polyhydroxyalkanoates, are the most attractive option [45]. A plethora of representatives of this class of polyesters of bacterial provenance find active application in biomedicine, with poly-3-hydroxybutyrate (PHB) being the most pervasive [46]. PHB is a semi-crystalline polymer. It is characterized by high biocompatibility and rapid conversion to oligomers and monomers under the influence of enzymes. These are then readily incorporated into the body’s metabolic pathways [47]. PHB’s unique supramolecular structure makes it easy to incorporate a variety of additives into it as a matrix. This ensures that the additives are located in the amorphous phase and, as a result, are released gradually as the hydrolysis process continues [48,49,50]. However, one of the simplest ways of administering antimicrobial additives is electrospinning of PHB. This is an effective method for creating a highly developed fibrous structure incorporated with additives with a possibility of the controlled rate of its release [51,52].

Electrospinning is one of the most striking examples of an approach to obtaining a highly developed hierarchical structure based on PHB [53,54]. Electrospinning is a versatile and viable technique for generating ultrathin polymeric fibers with the possibility of their modification by various components [55]. Moreover, there is a sufficient number of works where a successfully combination of PHB with different additives and components were shown and an improving of the characteristics of the polymer material, including increasing strength, chemical resistance or giving antimicrobial activity and other unique properties was detected [56,57,58,59]. In addition, a large number of works are known where low concentrations of polar molecules and nanoparticles are used, which also significantly improve the molding properties of PHB and, as a result, make it possible to obtain fibers in a wide range of characteristics of crystallinity, molecular weight, and surface microrelief [60,61]. Thus, electrospinning is of interest as an effective and easy way to incorporate additives of various nature into the PHB structure with the prospect of targeted modification of the properties of the polymer-additive system [62]. Moreover, electrospun systems allow to control the rate of release of substances, that allows not only to save the active agent and to control its consumption, but also significantly to increase the effectiveness of the system [63]. Of course, there are several limitations of the electrospinning method, such as low productivity or high degree of defect formation, which leads to low mechanical properties, but most of them could be solved due to new approaches in design od polymer-additives systems [64].

In our previous study on the creation of PHB-based chlorophyll extract delivery systems, which demonstrated greater effectiveness in suppressing the growth of pathogenic microorganisms than pure substance [65]. Therefore, further detailed study of PHB-Chl electrospun systems is an urgent task.

Herein we focused on obtaining comparative data on the antimicrobial activity of Chl-containing nettle extract, both in free form and as part of the PHB matrix, against Gram-positive and Gram-negative microorganisms using in vitro and in vivo models. In addition, new data on the nature of the mutual effect of Chl and PHB in the production of electrospun matrixes was obtained.

## 2. Materials and Methods

### 2.1. Materials

PHB powder (16F, BIOMER, Frankfurt, Germany); a nettle extract containing a Chl*a* and Chl*b* mixture (hereinafter referred to as Chl) [66]; analytical standard of chlorophyll *a* (≥95.0%, Merck, Darmstadt, Germany); analytical standard of chlorophyll *b* (≥95.0%, Merck, Darmstadt, Germany); analytical standard of oxytetracycline hydrochloride (OTC) (>98.0%, Merck, Darmstadt, Germany); dimethyl sulfoxide (DMSO) (99.5%, PanReac Applichem, Barcelona, Spain); chloroform (CL) Amresco (Solon, OH, USA); sterile physiological solution (0.9% NaCl, Khimikom, Nizhny Novgorod, Russia); meat-peptone agar (MPA, Khimikom, Nizhny Novgorod, Russia); acetone (≥99.75%, Ekos-1, Staraya Kupavna, Russia); dioxane-1,4 (≥99.5%, Ekos-1, Staraya Kupavna, Russia); salt meat-peptone agar (sMPA, Khimikom, Nizhny Novgorod, Russia); Endo agar (Biocontrol, Kazan, Russia); Sabouraud agar (Biocontrol, Kazan, Russia); meat-peptone broth (MPB, Khimikom, Nizhny Novgorod, Russia); the industry turbidity standard for determining total microorganism concentrations (BAK-10 kit, Ormet, Yekaterinburg, Russia); Zoletil 100 (Virba, Carros, France); Meditin (Apicenna, Moscow, Russia) were used in this work.

The chlorophyll-containing extract was obtained from the leaves of stinging nettle (*Urtica dioica*), collected in the Moscow region in August 2024. 23 g of dried and crushed stinging nettle leaves were heated in a drying oven (UT-4610, ULab, Beijing, China) for 2 h at 50 °C. Then 500 mL of 80% (*v*/*v*) aqueous acetone solution were added and vigorously stirred for 5 min at 800 rpm on a magnetic stirrer (DLab, model MS-H280-Pro, Beijing, China). Next, the extract was vacuum filtered using a vacuum pump (UV-2002, ULab, Beijing, China) through a Schott filter with a pore diameter of 16 μm. 50 mL of dioxane-1,4 cooled to 5 °C were added to the resulting extract. The extract was then cooled to 5 °C in a laboratory refrigerator (HL-250, POZIS, Zelenodolsk, Russia). 70 mL of ddH_2_O cooled to 5 °C was added dropwise to the extract. The extract was kept in the refrigerator for 2 days at 4 °C. The precipitate that formed was filtered using a vacuum pump through a Schott filter with a pore diameter of 5 µm. The Chl content was determined using a UV-Vis-spectrophotometer (PE5400UF, Ekroskhim, Saint Petersburg, Russia).

*Staphylococcus aureus* (strain 209-P), *Escherichia coli* (strain 1257) and *Bacillus cereus* (strain 96) were utilized. The strains were obtained from a cell culture collection maintained by the All-Russian Research Institute for Veterinary Sanitation, Hygiene, and Ecology.

### 2.2. Electrospinning of Chl-PHB

Forming solution were prepared by a standard technique [65]. Concentration of Chl were 0, 0.5, 1, 1.25, 1.5 wt. %. Concentration of PHB in CL was 7 wt. %. Fibers were obtained using EFV-1 ES scale with a single capillary (Moscow, Russia). Capillary diameter was 1 mm; temperature was 25 °C; the humidity was 38–40%; voltage was 16–20 kV; flow rate was 150–235 mL/min; distance between the electrodes was 200–220 mm. All materials were obtained from 25 mL of forming solution. Electrospun materials were kept in a desiccator until the complete evaporation of CL for 24 h at 25 °C.

### 2.3. Microscopy of Chl-PHB

Scanning electron microscopy (SEM) images were obtained by the Tescan VEGA3 scanning electron microscope (Tescan, Wurttemberg, Czech Republic). 1 cm^2^ of the Chl-PHB samples was covered with the platinum layer. Accelerating voltage was 20 kV.

Visualization of the Chl location was obtained by the confocal microscopy using Zeiss Axio Observer Z1 with a confocal module CSU-X1M 5000 with an oil lens (Carl Zeiss AG, Oberkohen, Germany).

### 2.4. Mechanical Properties

Mechanical properties of Chl-PHB samples were studied using a DVT GP UG compression testing machine (Devotrans, Istambul, Turkey). The strain rate was 5 mm/min (without preloading) according to the technique [67]. The size of the test sample was 10 × 40 mm. Tensile strength, elongation at break, and Young’s module were calculated automatically.

### 2.5. Differential Scanning Calorimetry

Differential scanning calorimetry (DSC) curves of Chl-PHB samples were obtained by the Netzsch 214 Polyma (Netzsch, Selb, Germany) in an nitrogen atmosphere according to the standard technique [68]. DSC program included 2 cycles: heating from 20 °C up to 200 °C and cooling from 200 °C to 20 °C with the rate 10 °K/min. Test samples was 5–7 mg. The enthalpy of melting and the melting temperature were calculated using Netzsch Proteus software according to the standard technique. The crystallinity degree was calculated according to the technique by the formula [69]:

(1) $$\chi =\frac{∆H}{H_{PHB}}\;\times \;100\%$$

where ∆H—melting enthalpy; $H_{PHB}$—melting enthalpy of the ideal crystal of the PHB (146 J/g) [70].

### 2.6. Electron Paramagnetic Resonance (EPR)

EPR-V automatic spectrometer (Moscow, Russia) was used for recording EPR spectra. The modulation amplitude was ≤0.5 G. The stable nitroxyl radical TEMPO was used as the spin probe. The experimental spectra of the spin probe in the region of slow motions (τ > 10^−10^ s) were analyzed within the model of isotropic Brownian rotation using the program described in [71]. Correlation time τ in the region of fast rotations (5 × 10^−11^ < τ < 10^−9^ s) was found by the formula [72]:

(2) $$\tau ={∆H}_{+}\;\times \;\sqrt{\frac{I_{+}\;}{I_{-}}}-1\times 6.65\times 10^{-10}$$

where ΔH_+_ is the width of the spectrum component located in a weak field and $\frac{I_{+}}{I_{-}}$ is the ratio of the component intensities in the weak and strong fields, respectively.

### 2.7. Fluorescent Spectroscopy

Fluorescence spectra of Chl in PHB were recorded using Cary-Varian 500E spectrophotometer (Agilent Technologies, Varian, CA, USA) in the range of 640 to 740 nm (UV-Vis range) [73].

### 2.8. Stock Solutions Preparation

The stock solutions of Chl-containing nettle extract (Chl) were prepared in DMSO to obtain the concentration of 1 mg/mL and stored at room temperature (22 ± 2 °C) without light.

The stock solutions of OTC were prepared using sterile physiological solution just prior to the experiments.

### 2.9. Irradiation Source

A LED lamp (Omega light, Seoul, Republic of Korea) was used as an irradiation source. The samples and animals were irradiated at 640 nm for 30 min (light dose—16.2 J/cm^2^).

### 2.10. In Vitro Chl Release from PHB

To investigate the release of Chl (nettle extract and mixture of substances) from PHB, 1 cm^2^ sample of polymeric material with 0.5%, 1.0%, 1.25% and 1.5% (% *w*/*w*) Chl content was placed in a tube containing 10 mM PBS with pH 7.4. PBS was added to each sample in the volume required to achieve the maximum concentration of released Chl—20 μg/mL. Previously, we established that at such a concentration of Chl, the optical density does not exceed 1. The concentrations of Chl in polymeric forms were as follows: 1.5%—180 μg/cm^2^; 1.25%—150 μg/cm^2^; 1%—120 μg/cm^2^; 0.5%—60 μg/cm^2^. These Chl concentrations were adjusted during the production of polymeric forms and further monitored by Chl extraction from the polymeric matrix with DMSO and subsequent spectrophotometry. The design of the Chl release study from the polymeric matrix was based on a generally accepted methodology [74]. The samples were incubated with constant stirring using a magnetic stirrer (DLab, model MS-H280-Pro, Beijing, China) for 72 h (200 rpm, 37 °C). The percentage of released Chl was determined by measuring the OD of test solutions using a PE5400UF spectrophotometer (Ekroskhim, Saint Petersburg, Russia) and QA5400 software (version 2.1, Ekroskhim, Saint Petersburg, Russia). The test solutions were collected every hour during the first 12 h of the experiment, then once every 12 h. The results represent the average value of measurements of three samples with standard deviation.

### 2.11. In Vitro Antimicrobial Activity Assessment

#### 2.11.1. Evaluation of the Minimum Inhibitory Concentrations (MICs)

To determine the MICs for Chl and Chl-PHB, test solutions with concentrations ranging from 250 to 0.95 μg/mL were prepared from the stocks using the serial dilution technique.

20 µL of each Chl solution and 180 µL of sterile liquid nutrient medium (MPB) contaminated with microbial suspension containing 10^8^ CFU/mL were added to sterile 96-well microplates (ServiceBio, Wuhan, China). Chl-PHB fragments with equivalent active substance contents were placed into 48-well plates (ServiceBio, Wuhan, China). Some plates were exposed to specific irradiation conditions, while others were not irradiated to investigate the light-independent effects.

The samples were incubated for 24 h at 37 °C with stirring on an orbital shaker (Joanlab, model SK-20, Huzhou, China) at 80 rpm. OD was measured using a microplate reader (Mindray, model MR-96A, Shenzhen, China) at 600 nm.

The control group consisted of cell suspensions that were not subjected to any prior treatment, but were supplemented with solvents such as DMSO or ddH_2_O. The minimum inhibitory concentration (MIC) was determined as the concentration at which no change in optical density (OD) occurred compared to the value obtained before incubation. All experiments were performed in triplicates.

#### 2.11.2. The Microorganism Growth Inhibition Degree Calculation

In order to assess the inhibitory effect of various concentrations on *S. aureus*, *E. coli*, and *B. cereus* both under irradiated and non-irradiated conditions, we measured the OD_600nm_ of experimental samples containing pathogens and preparation solutions, as well as control samples consisting of sterile medium, medium with preparations but without pathogens, and medium without preparations. The inhibition of microbial growth (as a percentage) was calculated using the following formula:

(3) $$1-\;\frac{{OD}_{test\;solution}\;-\;{OD}_{corresponding\;control}}{{OD}_{assay\;growth\;control}\;-\;{OD}_{sterility\;control}}\;\times 100$$

#### 2.11.3. Inhibition Zone Diameters (IZDs) Determination

The determination of the antibacterial activity of Chl and its polymeric forms was conducted using a dense nutrient medium (MPA) by measuring the diameter of the inhibition zones of growth (IZDs) formed around wells containing the Chl solutions and Chl-PHB fragments. Initially, 1 mL of a microbial suspension with a concentration of 10^5^ colony-forming units per milliliter (CFU/mL) was inoculated into sterile MPA Petri dishes. Wells were subsequently created in the center of each MPA plate using a sterilized punch, and 100 µL of the solutions were introduced into each well. Fragments of Chl-PHB, prepared at concentrations of 0.5, 0.75, 1.25, and 1.5 percent by weight relative to the weight of PHB, measuring 1 square centimeter in area, were positioned in the center of the inoculated Petri dishes.

In order to investigate the photodynamic activity of the test samples, some Petri dishes were exposed to irradiation using a LED lamp as described in Section 2.9. To prevent any potential interference of light with the experimental outcomes, all procedures were conducted in a space with diffuse ambient lighting, devoid of any supplementary light sources.

Next, the samples were placed in an air-dry thermostat (TV-80, Kasimov Instrument Plant, Kasimov, Russia) and incubated at 37 °C for 120 h with daily recording of IZDs (mm) using a digital caliper (ADA Mechanic, model 150 A00379, Hong Kong, China). The experiments were performed in triplicate.

#### 2.11.4. Phase Contrast Transmission Electron Microscopy (TEM)

0.5 mL of microbial culture (10^9^ CFU/mL) was added to a test tube with 5 mL of sterile MPB. The test tube was placed in a thermostat at 37 °C to increase the bacterial mass. 100 µL of the preparations (2 × MIC dose) were added to the test tubes. Some samples were irradiated under a LED lamp for 30 min. The control sample was not exposed to preparations and irradiation.

Next, incubation was carried out for 12 h in a dry air incubator at 37 °C. 1.75 mL of each sample was placed in test tubes and centrifuged at 14,500 rpm for 15 min in a centrifuge (Eppendorf MiniSpin plus, Eppendorf, Hamburg, Germany). The supernatant liquid was removed. 1.75 mL of sterile ddH_2_O was added to the sediment. The samples with dense sediment were placed in an ultrasonic bath (Sapfir, AZ Engineering, Moscow, Russia) for 20 min at 25 °C. Then the centrifugation cycle was repeated to remove the supernatant. 0.5 mL of sterile ddH_2_O was added to the resulting centrifugate and shaken on a vortex mixer (Vortex V-1 plus, BioSan, Riga, Latvia) until a homogeneous suspension was obtained.

The samples were thoroughly mixed on a vortex for 10 s, then 5 μL of each sample was applied to a TEM grid for 3 min. Then, the liquid phase was removed, and the grid with the fixed sample was treated with 5 mL of a 2% aqueous solution of sodium uranyl acetate for 3 min. After repeated removal of the liquid phase, the grid was placed in a TEM (JEM-2100 plus, JEOL Ltd., Tokyo, Japan). Then imaging was performed at a direct magnification of 8000×, 650 ms exposure using NANOSPRT43 camera (JEOL Ltd., Tokyo, Japan).

### 2.12. Assessment of Wound Healing and Antimicrobial Activity in Mouse Model

Eight-week-old and weighing 20 ± 2 g male Balb/c mice (Scientific Center for Biomedical Technologies of the Federal Medical and Biological Agency, Stolbovaya branch, Russia) were used for the experiment.

The animals were quarantined for 14 days before the experiment began. The mice were maintained under a consistent 12 h light-dark cycle, with unrestricted access to food and water. The method of random sampling was employed to divide the mice into nine groups, each comprising eight individuals (Table 1).

To initiate wound formation, animals were anesthetized intraperitoneally with Zoletil 100 and Meditin at doses of 12.5 and 1 mg/kg, respectively. Then the hair was shaved off in the middle of the back and the skin was treated with 70% (*v*/*v*) ethanol. The resection of a skin area of approximately 2 cm^2^ was performed using sterile surgical scissors. To achieve purulent inflammation, 100 μL of *S. aureus* strain 209-P bacterial suspension containing 10^9^ CFU/mL were introduced into the wounds.

Treatment was started 48 h after infection, when the development of purulent inflammation in the wounds was obvious. Animals were treated every day for 7 days. 100 μL of Chl and OTC solutions was added into the wounds at the indicated dosage. DMSO was added in the same volume. Chl-PHB and Chl-free PHB fragments of 2 cm^2^ were applied to the wound and secured with adhesive tape. Animals in the photodynamic effect study groups were irradiated for 30 min with 16.2 J/cm^2^ light dose.

During the experiment, the general condition and physical activity of the animals, as well as the appearance of the wound, were visually assessed. The wound surface area was calculated using the formula:

(4) S=L×W×0.785

where L is the length of the wound; W is the width of the wound; 0.785 is a constant [75].

The wound surface was washed with sterile swabs on the 7th day of the experiment. 1 mL of the washes was cultured on saline MPA, Endo agar and Sabouraud agar to isolate *Staphylococcus* spp., *Enterobacteriaceae* spp. and microscopic fungi, respectively. Bacterial cultures were incubated in a thermostat for 48 h at 37 °C, while microscopic fungi were cultured for 168 h at 28 °C. Colonies were counted daily using a Scan 3000 AI automatic counter (Interscience, Cantal, France).

The experiment was conducted in strict adherence to the regulations outlined in Federal Law No. 498-FL “On Responsible Treatment of Animals” (Russian Federation, 2018), as well as Directive 2010/63/EU, issued by the European Parliament and the Council of the European Union, concerning the protection of animals utilized for scientific purposes. Additionally, the research was guided by the “Guide for the Care and Use of Laboratory Animals”, published by the National Academy Press in the United States (2011) and the “Guidelines for Conducting Preclinical Studies of Medicines”, issued by the Russian Federation (2012).

The protocol for this study was reviewed and approved by the Bioethics Committee of the All-Russian Research Institute for Veterinary Sanitation, Hygiene, and Ecology (protocol number: 01/25-RRIVSHE, issue data 30 April 2025).

### 2.13. Statistical Analysis

The data analysis was carried out via GraphPad Prism 10.4.0.621 Software (GraphPad, San Diego, CA, USA), using one-way multiple comparison analysis of variance (ANOVA). Dunnett’s test was used. Statistical significance was accepted at a threshold of *p* < 0.05. All the measurements were performed in triplicate. The results are presented as means (M) and standard deviation (±SD).

## 3. Results and Discussion

### 3.1. Characterization of Chl-PHB Matrix

**Figure 1:**
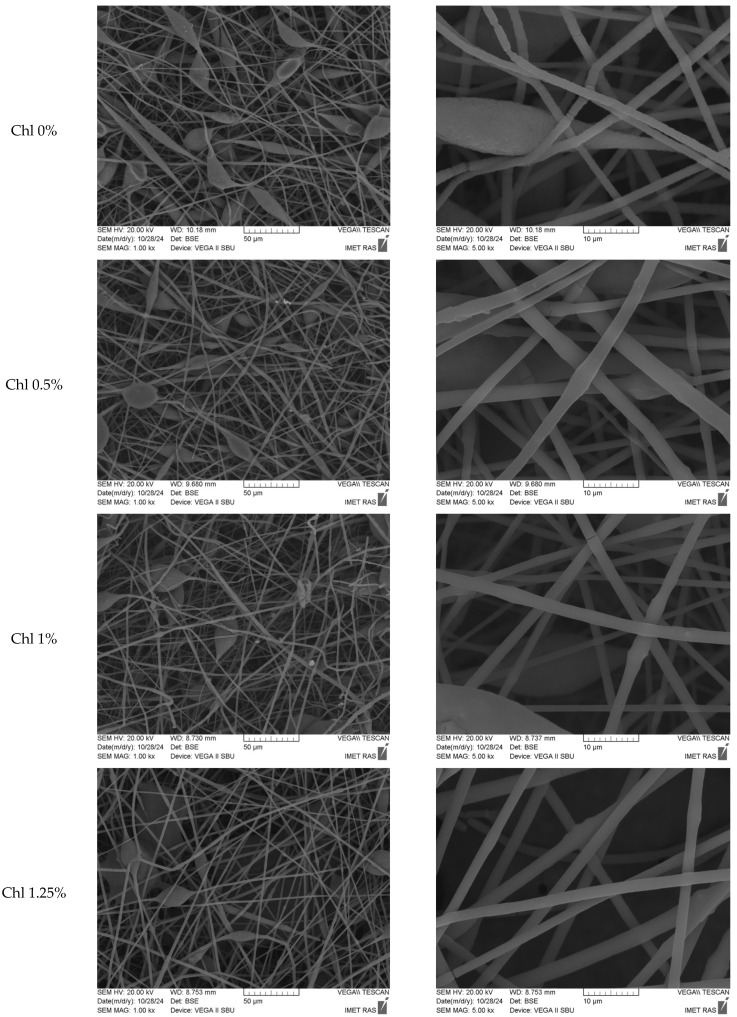

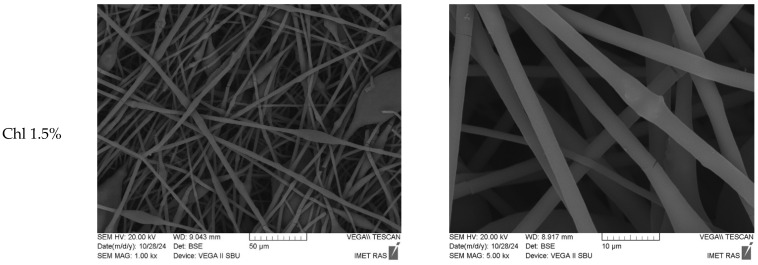
SEM images of Chl-PHB matrixes.

Figure 1 shows microphotographs of fibrous materials based on PHB-Chl. The fibers have non-uniform morphology. They are characterized by the presence of smooth cylindrical sections. There are also arbitrarily shaped thickenings. An average diameter of 2.9 μm is possessed by the smooth sections of the fibers. Thickening sizes typically range from 35 to 50 µm on average. Thickening may be due to the low polarity of PHB, resulting in an uneven distribution and flow of charge during fiber formation. This happens at the stage of the primary jet of the solution [48].

The solution’s stability is evidenced by the absence of finer fibers in the structure of nonwoven fibrous material, as no splitting of the primary jet occurs during the forming process. The integration of additional polar chlorophyll molecules into the fiber formulation results in enhanced charge run-off and the standardization of the electroforming process. It is important to mention that thickenings is a type of fibrous defect which is often observed during electrospinning of PHB [76,77]. Such defects can be easily eliminated by introducing additional components plasticizers, co-solvents or additives especially polar molecules [78,79,80].

**Figure 2:**
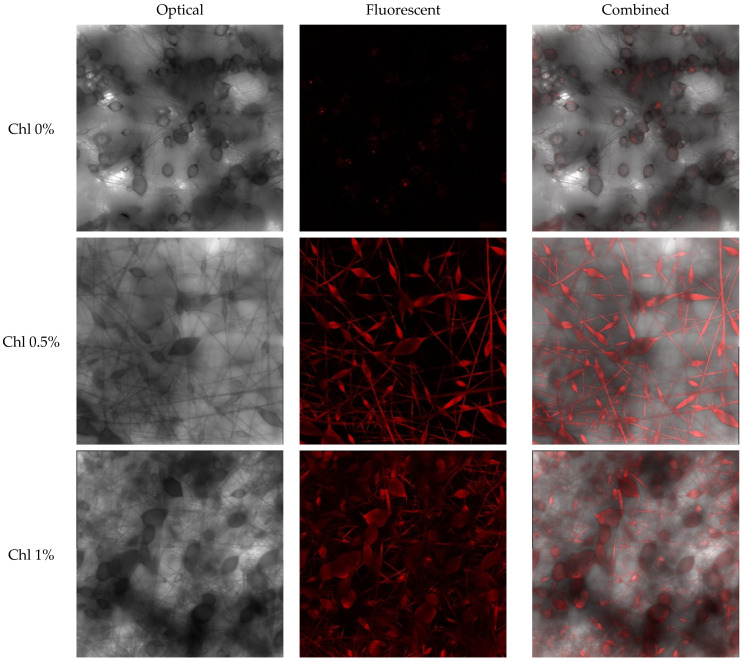

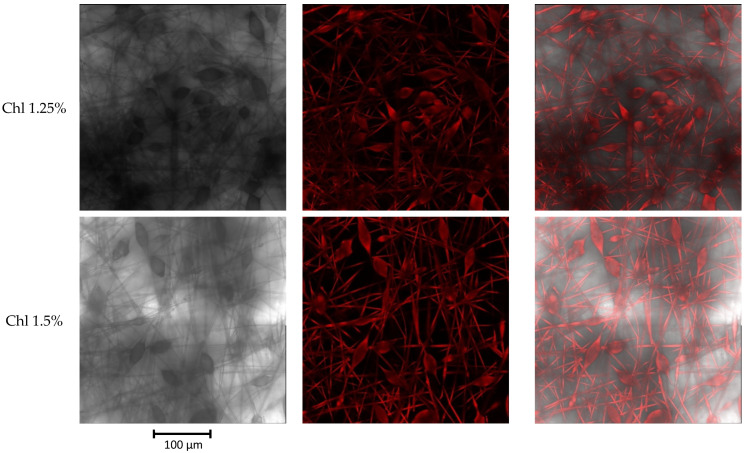
Confocal microscopy images of Chl-PHB matrixes, 40 × magnification.

Concurrently, the mean diameter of the thickenings on fibers diminishes to 25–30 μm, and there is a propensity for a slight augmentation in the mean diameter of cylindrical fibers with an increase in the concentration of Chl up to 1.5%. Notwithstanding the augmentation in the mean diameter, the incorporation of the additive material effectuates a substantial diminution in the incidence of fiber imperfections, manifesting as thickenings on the fiber’s surface. Chl facilitates the acquisition of a material with elevated levels of porosity and surface development, devoid of underflows and adhesions. Fluctuations in the average fiber diameter can be explained by the effect of low Chl concentrations and intermolecular interactions with PHB, as demonstrated by the fluorescence spectroscopy data presented below. Substances with low molecular weight and low concentration may not be spread evenly through the fibers. To perform a more comprehensive examination of Chl distribution within the fiber volume, we employed confocal microscopy (Figure 2).

Confocal microscopy results, in which fluorescent molecules are highlighted in red, demonstrate the uniform distribution of Chl in the PHB matrix. It is evident that the luminescence intensity rises in proportion to the concentration of Chl.

The additive is spread evenly throughout the whole structure of the PHB at a concentration of 1.5%. At lower concentrations, it is more likely to be found in defect areas, where it becomes thicker. Increased concentration of Chl in the fibers is shown to be associated with a tendency for increased density of the weave network and interlocking nodes, as revealed by a closer analysis of the micro-photographs. This morphology can significantly impact the strength of nonwoven fiber materials.

The mechanical strength, modulus of elasticity and relative elongation of PHB-Chl fibrous materials under uniaxial tension are shown in Table 2.

As shown in the table, the addition of Chl to PHB increases the average elastic modulus and tensile strength, while reducing the relative elongation at break. This conduct of the substances is corroborated by the establishment of a more substantial framework in the form of a close-knit network of physical fastenings and knots of fiber interlacing during the creation of nonwoven substance on the deposition electrode. Evidence for this has been provided by the data of confocal optical microscopy. The random distribution of fibers on the substrate of the deposition electrode leads to a non-uniform flow of electric charge from the fibers surface. This, in turn, causes fluctuations in the values of mechanical indices. The reason for this is a small gradient of the density of the nonwoven fibrous material layer.

Concurrently, a propensity to diminish the strength and elastic modulus of fibrous materials with an escalating concentration of Chl is discernible. This behavior can be explained by the supramolecular structure of the fibers. The supramolecular structure of fibers was investigated using DSC and EPR methods.

**Figure 3:**
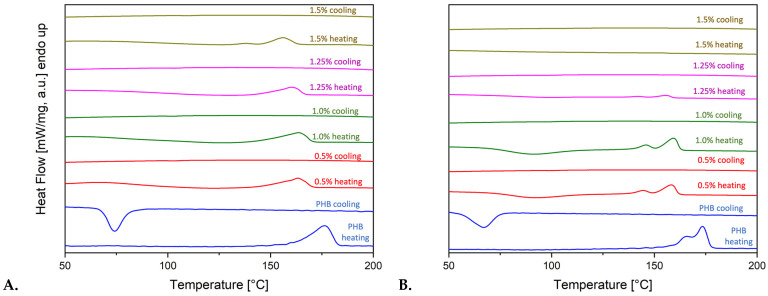

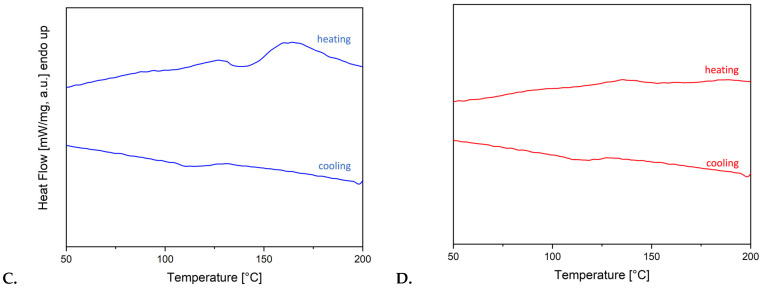
DSC curves of Chl-PHB matrixes and pure Chl, where (**A**)—1 heating-cooling cycle of PHB-Chl matrixes; (**B**)—2 heating-cooling cycle of PHB-Chl matrixes; (**C**)—1 heating-cooling cycle of Chl; (**D**)—2 heating-cooling cycle of Chl.

The acceleration of the crystallization of PHB by Chl is clearly visible in the DSC curves (Figure 3A,B), regardless of concentration. The addition of Chl to the PHB matrix results in a substantial increase in the crystallization temperature (Table 2). This indicates sufficiently strong intermolecular interactions between the components of the mixture. As can be seen from the structural formulas, intermolecular bonds can be formed by PHB and Chl through the mechanism of hydrogen bond formation due to the presence of polar groups. Concurrently, with the augmentation of the Chl content in the system, the indications of the imperfection of the formed crystal structure are observed.

DSC curves of pure Chl extract is shown in Figure 3C,D. Chlorophyll *a* has a melting point of between 150 and 153 °C, while chlorophyll *b* has a melting point of 120 °C [81]. However, in the case of the extract, the experimental temperatures are slightly higher: 127 for Chl and 160 °C for Chl*a*. The division into two fractions is clearly visible on the DSC curves and correlates with the ratio of 3:1 of Chl*a*–Chl*b*. This means that the melting peak occurs earlier as the chlorophyll concentration increases, and this may be related to the temperature effects of the additive. Thermo-physical properties of Chl-PHB matrixes are shown in Table 3. Secondary melting also leads to the observation of intermolecular interactions between components in the mixtures. There is a severe fall in the melting, crystallization temperatures and enthalpy of melting of PHBs. When the second heating is applied, the melting peak separates into two groups of crystallites: normal and smaller crystallites. This is due to the effect of Chl. As the amount of Chl goes up from 0.5% to 1.5%, the PHB does not turn into crystals when the mixture is reheated. This is due to the increased viscosity of the PHB in its molten state. Crystallization processes after the first melting, resulting from intermolecular interaction, are characterized by delay and non-equilibrium. These processes lead to cold crystallization during secondary heating, as evidenced by exo-thermal peaks in the temperature range of 75–100 °C on thermograms of repeated melting.

**Figure 4:**
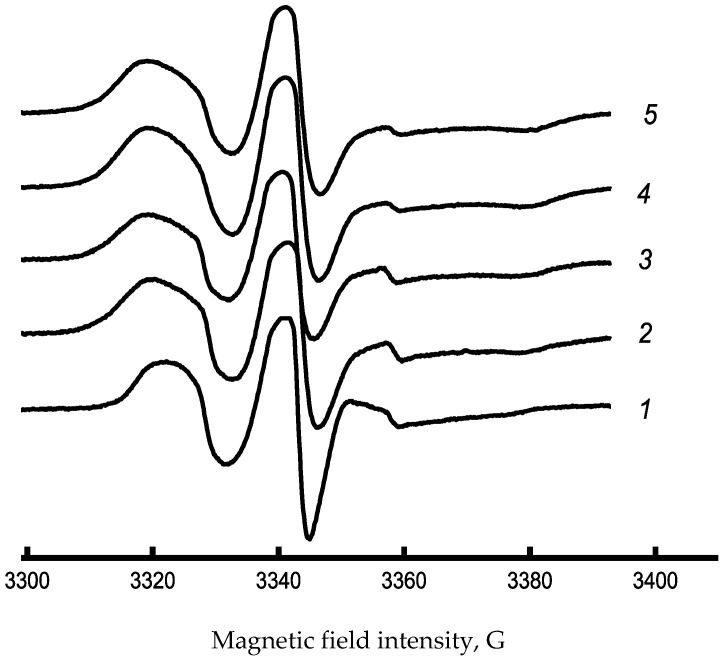
EPR spectra of TEMPO spin probe Chl-PHB matrixes, where 1—0%, 2—0.5%, 3—1.0%, 4—1.25%, 5—1.5% of Chl.

So, to describe the supramolecular structure in the amorphous regions of PHB-Chl fibers, we used the EPR probe method. Typical EPR spectra of blended PHB-Chl fiber materials are shown in Figure 4. These spectra reveal an increase in the shoulder region between 3320 and 3330 G. The shift in the spectrum’s characteristics could be a consequence of the intermolecular interaction between PHB and Chl, resulting in the consolidation of the amorphous phase. The correlation time increases significantly with increasing Chl concentration, as shown in Table 4. This indicates that the structure of PHB become more compact in the amorphous regions as the concentration rises.

The 1% point is an interesting exception to the general trend, both in terms of crystallinity and correlation time. From the investigation of the structure of the crystalline and amorphous parts of PHB-Chl fibers, it can be deduced that the tensile strength of nonwoven fibrous materials reduces due to a reduction in the degree of crystallinity and compaction of the amorphous phase as a result of the creation of intermolecular bonds. The fiber’s transformation into a more amorphous and rigid state leads to a decrease in its strength. However, the development of physical mesh and weave knots ensures that the nonwoven material maintains its high physical and mechanical properties.

Summarizing the data obtained on the role of chlorophyll in the formation of the supramolecular structure of PHB, the photosensitizing nature of the additive should be mentioned, which improves the morphology of the fibers, but leads to nonlinear changes in the supramolecular structure of PHB. It could be explained by local aggregation in the amorphous phase of the polymer, which is especially noticeable in the EPR results. At the same time, the changes in the crystalline phase occur rather linearly and demonstrate that the additive prevents the crystallization of PHB.

Tetrapyroles and their derivatives often have a positive effect on the supramolecular structure of PHB, improve forming properties for electrospinning, and as a result, the morphology of the fibrous layer, increasing mechanical characteristics [74,82]. However, in some cases, when the photosensitizing properties are too high or the size of the molecule is large enough, which makes crystallization difficult, the opposite effects are possible [83]. The data obtained clearly showed that chlorophyll concentrations of more than 1% lead to nonlinear changes in the strength and structure of the amorphous phase, and sharply reduce the proportion of crystallinity. Previously the FTIR spectra of PHB-Chl were obtained [65]. It was found that chlorophyll is located on the surface of the fibers, and its amount is proportional to the concentration, as evidenced by an increase in the intensity of FTIR peaks of the most pronounced chemical groups. The formation of a hydrogen bond between PHB and Chl is quite likely due to the presence of the carbonyl group of PHB and amino groups in Chl [84,85]. In addition, the previously obtained data show that the hydrophilicity of the material increases with increasing Chl concentration, and the appearance of additional -OH also contributes to better bonding of the additive and polymer due to hydrogen bonds.

### 3.2. Fluorescence of Chl-PHB Matrix

**Figure 5:**
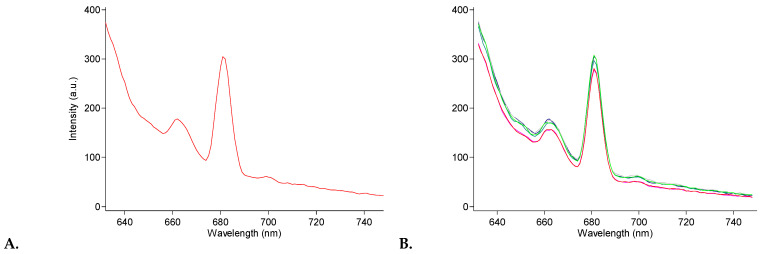
Fluorescence of Chl-PHB matrixes, where (**A**)—0.5%, (**B**)—all samples of Chl-PHB.

Fluorescence of Chl-PHB matrixes is shown in Figure 5. All nonwovens showed a similar fluorescence pattern, which matched the absorption pattern of the chlorophyll *a* and *b* mixture. The maximum of chlorophyll *a* was around 650 nm, while chlorophyll *b* was around 670 nm [49]. However, we noticed a slight red shift at 660 nm and 680 nm, respectively. This transition may be attributed to the interaction of molecules with the polymer, though it is noteworthy that its significance is negligible in this instance.

### 3.3. In Vitro Chl Release from Polymeric Matrix

**Figure 6:**
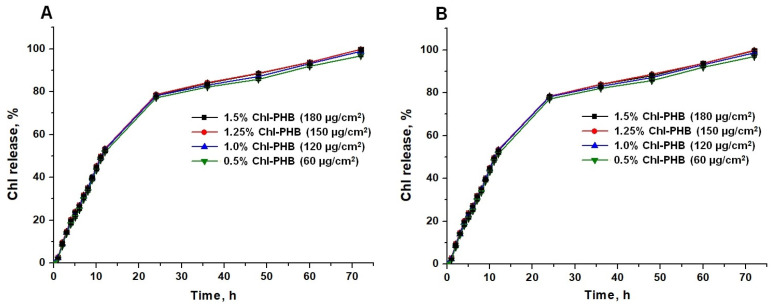
Mixture of Chl*a* and Chl*b* substances (3:1) (**A**) and Chl-containing extract (**B**) release profile from PHB at 37 °C and pH 7.4 in 10 mM PBS. *p*-value is <0.05.

To evaluate the prolonged action of the developed polymeric form, the release of a mixture of Chl*a* and Chl*b* substances (3:1), as well as a Chl-containing extract from PHB was studied. The study showed that in both formulations, the kinetic release profiles were identical regardless of the Chl concentration in the carrier (Figure 6A,B).

It is evident that Chl exhibited an exponential release pattern followed by a slow phase. Within the first 24 h, depending on the specific sample, a rapid release of Chl was observed, ranging from 77.07% to 78.35% for the Chl-containing extract and from 77.14% to 78.72% for the Chl mixture. Thereafter, a phase of steady and slow release commenced. A near-complete release of the active substance was achieved, ranging from 96.91% to 99.83% for the Chl-containing extract and from 96.75% to 99.79% for the Chl substances mixture, by 72 h. The initial burst in Chl release can be attributed to the presence of weak non-covalent interactions between the molecules of the substance and the polymeric matrix.

It is obvious that in order to maintain an effective concentration of biocides intended for the treatment of wound infections, their controlled release over several hours or even days is necessary. It ultimately allows interrupting the logarithmic phase of microorganisms’ growth [86,87]. In this regard, satisfactory results have already been obtained in materials with different polymeric structures [88,89,90]. Herein, we developed polymeric system based on biodegradable polymer PHB for the treatment of infected wounds. Considering the relatively high rate of microbial reproduction, the determined release time of Chl can be considered adequate for addressing this issue. Moreover, the obtained data serve as a valuable foundation for further assessment of the antimicrobial activity of Chl-PHB both in vitro and in vivo. By analyzing the timing and proportion of biocide release from the matrix, we can anticipate its concentration at the site of application.

### 3.4. In Vitro Antimicrobial Activity of Chl and Chl-PHB

In current studies, we investigated two pathways of Chl and Chl-PHB activity: light-independent and under irradiation. Representatives of Gram-positive microorganisms—*S. aureus* and *B. cereus*, as well as Gram-negative ones—*E. coli* were selected as test cultures. *S. aureus* is part of the normal skin microbiota and *E. coli* can be found in contaminated wounds. Against the background of a decrease in the host organisms resistance, both microorganisms cause severe inflammation [91,92]. *B. cereus* can also infect contaminated wounds as it is present in the soil in both vegetative and spore forms. In addition, *Bacillus* is often found in gunshot and stab injuries [93].

#### 3.4.1. MICs Determination Results

At the initial stage of microbiological test, MICs were determined to evaluate the antimicrobial potential of Chl preparations. The data obtained are presented in Table 5.

As can be seen from the data presented, Chl and Chl-PHB exhibited light-independent and photodynamic antimicrobial activity against the tested microorganisms. The effect of these compounds on *E. coli* was approximately three times less than that on *S. aureus*, while *B. cereus* appeared to be more susceptible to the effect of Chl in both its forms. Irradiation appeared to have a positive effect on the MICs of the compounds. The inhibitory activity of the preparations increased by approximately twofold when treated with *S. aureus* and *E. coli*, and by fourfold for *B. cereus*.

The results obtained are consistent with our previous data, where the Chl’s MIC for *E. coli* was three times higher than for *S. aureus*. The more pronounced resistance of Gram-negative bacteria to biocides can be explained by the peculiarity of the cell wall structure, which causes low permeability of molecules [94,95,96].

#### 3.4.2. Study of Microorganism Growth Inhibition

In a previous investigation of MICs, it was discovered that microbial growth resumed two to five days after exposure to specific concentrations of Chl and Chl-PHB, both with and without irradiation. This finding suggests the lack of a bactericidal effect, indicating that the use of MICs does not guarantee the complete destruction of microorganisms, rather, it merely reduces the number of viable cells. The same conclusion was made by Blondeau J.M. et al. when studying various MIC multiplicities of a number of drugs [97]. Therefore, in the current study, we explored the feasibility of achieving a sustained bacteriostatic response under the influence of a dual MIC (Table 6).

As follows from the data presented, over 99 percent of microorganism growth inhibition was observed when exposed to doses of preparations exceeding 2 × MIC. An irradiated Chl-PHB demonstrated the highest level of inhibitory activity. Thus, dosages from 2 × MIC and above can be considered promising for future studies.

#### 3.4.3. Inhibition Zone Diameters (IZDs) Evaluation

**Figure 7:**
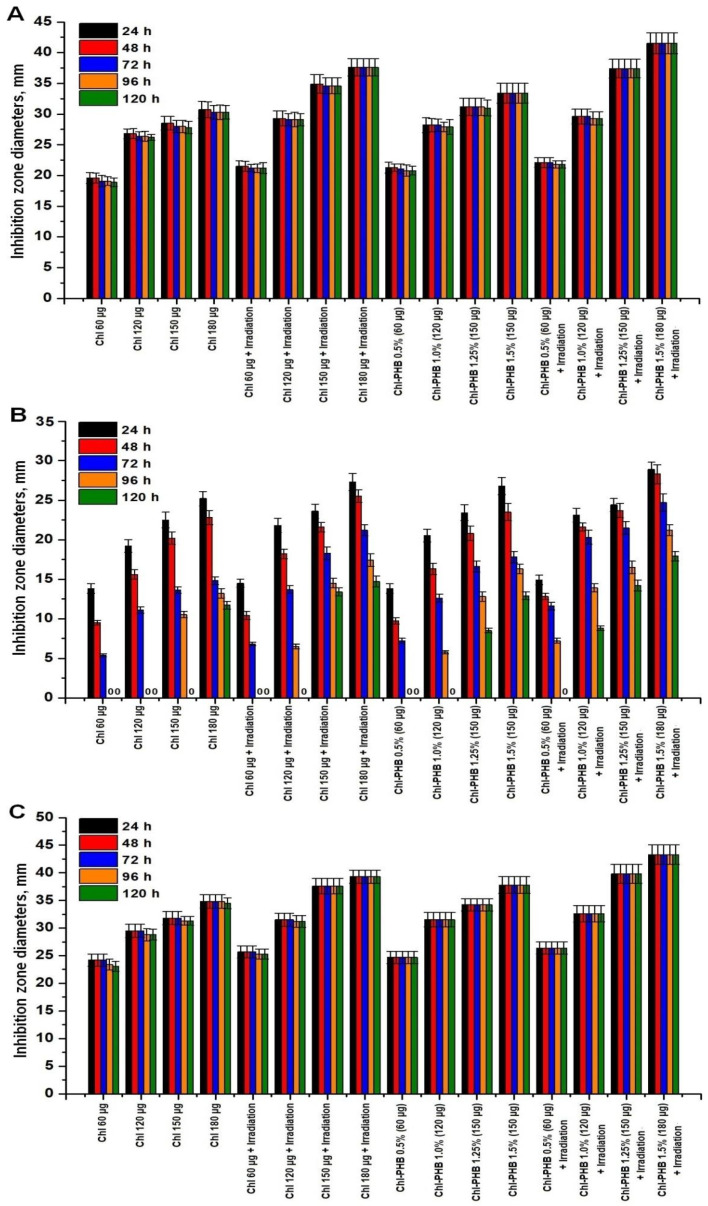
IZDs values of Chl and Chl-PHB against *S. aureus* (**A**), *E. coli* (**B**) and *B. cereus* (**C**) with irradiation and without it. *p*-value is <0.05.

In the next phase, the inhibitory effects of the preparations against *S. aureus, E. coli*, and *B. cereus* were evaluated by altering IZDs. The non-toxicity of PHB and DMSO had previously been demonstrated in our studies. The results of IZDs measurements for 120 h following the introduction of the preparations are presented in Figure 7.

The inhibitory effect on staphylococcus growth began to decrease at all doses of non-irradiated Chl from the third day of the experiment. However, the reduction in the IZDs did not exceed 3.5% relative to the values of the 1st day. The reduction in the IZDs during irradiation did not exceed 1.5% for doses of 60–150 μg. At a dose of 180 μg, the IZDs did not change over 120 h. The study of the activity of Chl-PHB revealed a dose-dependent effect on *S. aureus*. When *S. aureus* was exposed to preparations containing 1.5% Chl, stability of the inhibition zones was observed. Irradiated Chl-PHB with 1.25% active substance exhibited a persistent antimicrobial effect throughout the entire observation period. In contrast, non-irradiated Chl retained its activity against *E.coli* only at the highest dosage. Exposure to irradiation enhanced both the potency and the duration of action of both free form and carrier-associated Chl. Among the irradiated samples, the polymeric complex containing 1.5% of Chl demonstrated the highest efficiency. On the fourth and fifth days of observation, there was a slight increase in bacillus growth under the influence of non-irradiated active substances in dosages of 60, 120, and 150 μg. However, the IZDs of all irradiated Chl-PHB samples and the free form of Chl at dosages of 150 and 180 μg remained unchanged. Therefore, the incorporation of Chl into the polymer and subsequent irradiation treatment increased the inhibitory efficiency by 35.2%, 14.7% and 24.4% against *S. aureus*, *E. coli* and *B. cereus*, respectively.

Thus, the obtained data showed that the diameters of the growth inhibition zones were directly dependent on the dose of the active substance. Moreover, an irradiation increased the efficacy of Chl both in free form and into the carrier. The similar data demonstrating the dose-dependent effectiveness of the drugs, as well as the enhancement of the inhibitory effect even against resistant Gram-negative bacteria under the influence of irradiation, are presented in the works of Hurst A.N. et al. [98], Gonçalves A.S. et al. [11]; Baek Ki Ho et al. [99]; and Anguluri K. et al. [100].

#### 3.4.4. Phase Contrast TEM Results

**Figure 8:**
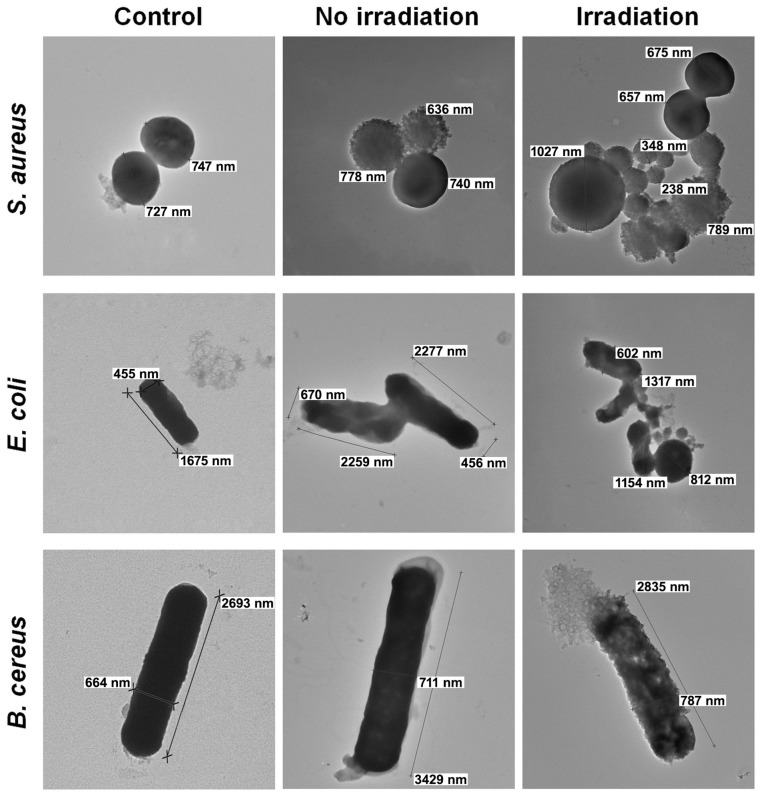
Phase contrast TEM images of *S. aureus*, *E. coli* and *B. cereus* incubated with non-irradiated and irradiated Chl (8000× magnification).

To study the influence of Chl on microbial cells, an assessment of their morphology changes was carried out using phase contrast TEM (Figure 8).

As can be seen in the images, intact microbial cells (the control) have a characteristic shape and size with clear edges and a smooth surface. In contrast, the texture of microorganisms treated with Chl at a dosage of 2 × MIC was rough, and the shape of the cells had changed. *S. aureus* cells appeared deformed and adhered, forming conglomerates, while *E. coli* and *B. cereus* showed an outflow of cytoplasm, indicating a violation of cell wall integrity. Under irradiation (16.2 J/cm^2^ for 30 min), the destructive processes were more prominent, suggesting that the mechanism of Chl’s action is associated with its ability to irreversibly damage the microorganisms’ cell walls. Irradiation undoubtedly enhances this effect, leading to the observed changes in the microorganisms’ morphology. A similar effect of irradiation on the cell wall of Gram-positive and Gram-negative microorganisms was noted in the works of X. Wang et al. [101], A.S. Garcez et al. [102], and R.K. da Silva et al. [103].

### 3.5. In Vivo Chl and Chl-PHB Wound Healing Evaluation and Antimicrobial Efficacy

The wound healing process is directly related to the immune system of the human and animals. A violation of skin integrity triggers the immune defense mechanism [104,105]. However, colonization of the skin by *Staphylococcus* spp. may become a complicating factor. These microorganisms are a part of the normal skin microbiota and are considered to be the main cause of wound infections and the development of purulent inflammation [92,106,107,108].

Chl has good prospects for combating staphylococcosis, as it is a photosensitizer for producing highly reactive singlet oxygen (^1^O_2_), which causes oxidative stress in microbial cells [43,109,110]. On the other hand, I. Stojiljkovic et al. reported the ability of metal-containing macroheterocycles to penetrate microbial cells and generate reactive oxygen species without irradiation [111]. Hence, a light-independent mechanism of action of Chl can be assumed. It has been confirmed in vitro in our previous studies.

In this study, Chl-PHB with a maximum biocide concentration of 1.5% (180 µg of Chl per 1 cm^2^) was utilized to assess antimicrobial activity in a murine model of infected wounds. OTC, an antibiotic from the tetracycline class, was employed as a control due to its strong activity against a variety of microorganisms, including Staphylococcus microbiota [112,113]. The DMSO was tested as a Chl’s solvent. Also, the potential contribution of Chl-free PHB to the antimicrobial activity was examined.

**Figure 9:**
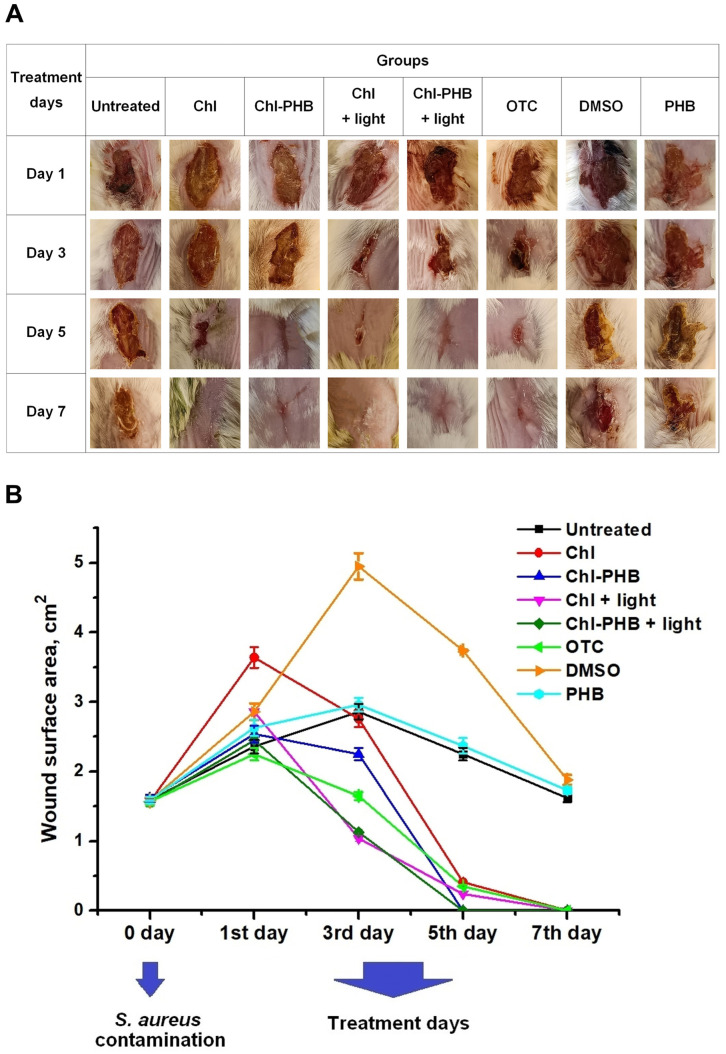
(**A**) Photos of *S. aureus*-infected wounds of mice groups on treatment days 1, 3, 5, and 7. (**B**) Changes in wound surface area under the influence of preparations. *p*-value is <0.05.

The wound healing process was monitored photographically throughout the entire treatment period (Figure 9A). Before treatment began, the wound inflammation of in all groups was typical and was characterized by pronounced erythema and swelling. The appetite and physical activity levels in animals were reduced.

On the 3rd day of treatment, in all groups other than Untreated, DMSO, and PHB, the behavior of the animals was not significantly different from that of the intact mice. No purulent exudate was observed in the wounds, but scab formation occurred.

The reduction in wound area relative to day 1 of treatment initiation was 31.88%, 12.9%, 63.65%, 53.87%, 26.67% for Chl, Chl-PHB, Chl + light, Chl-PHB + light, and OTC groups, respectively. In contrast, in the Untreated, DMSO and PHB groups, wound area increased by 21.18%, 73.07% and 12.55%, respectively (Figure 9B).

The 5th day of treatment was characterized by tissue regeneration in Chl, Chl-PHB, Chl + light, Chl-PHB + light, and OTC groups. An activity and mobility, as well as water and food consumption, were typical for mice. Significant reductions in wound area were observed: 88.78%, ~100%, 91.6%, ~100%, 84.44% for Chl, Chl-PHB, Chl + light, Chl-PHB + light, and OTC groups, respectively. In the irradiated groups, the wounds were covered with granulation tissue at the area of scab’s rejection. In the Untreated group, wounds began to close with a scab, their area decreased by 4.9%. The wounds in the DMSO group still secreted purulent exudate. The area reduction was 42.3% compared to the 3rd day. The wound condition in the PHB group was comparable to that of the Untreated group. The area of wounds decreased by 9.88%.

On the 7th day, in all groups with Chl, as well as the antibiotic, granulation tissue was replaced by epithelium. The scab size in the Untreated and PHB groups decreased by 45.67% and 34.22%, respectively. Despite the lessening in wound area in the DMSO group (by 34.26%), the purulent exudate secretion did not complete.

Thus, Chl preparations showed comparable efficacy with OTC. Nevertheless, the irradiation accelerated wound closure and skin regeneration. These results are consistent with the data of Chepurnaya J.L. et al., Wei He et al., Jiaying Li et al., and Ye He et al., which demonstrated a positive effect of irradiation on the course of the wound process [114,115,116].

**Figure 10:**
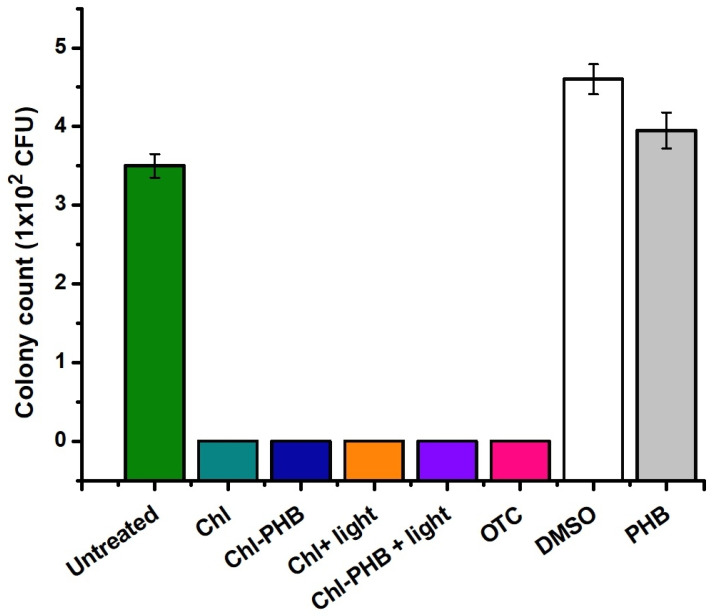
*S. aureus* colony counts on saline MPA (after 48 h incubation). *p*-value is <0.05.

On the 7th day of treatment, to study the in vivo antimicrobial activity of Chl and Chl-PHB, bacteria from infected mouse wounds were cultured for 48 h, and microscopic fungi for 168 h (Figure 10).

After two days of incubation in saline MPA, inoculated with washings from the Untreated, DMSO and PHB groups, active growth of *S. aureus* colonies was observed. Interestingly, the highest number of colonies was seen in the DMSO group, which is likely due to the increased tissue permeability caused by DMSO [117]. This resulted in more active staphylococcus penetration into the wound, leading to extensive inflammation. Additionally, it is possible that the property of DMSO enhances the effectiveness of Chl solutions. There is already evidence that DMSO facilitates transdermal delivery of drugs from various therapeutic groups. For example, A. Otterbach and A. Lamprecht noted that DMSO significantly improved the penetration of estradiol included in a transdermal patch [118]. The same effect was observed by J.J. Tarrand et al. when applying DMSO-containing antiseptic to the skin [119]. Since DMSO is able to penetrate not only through the lipid layer, but also through the subcutaneous adipose tissue, it can be assumed that its effect is preserved in damaged skin. In groups treated with Chl-based preparations or antibiotic, microbial growth was completely inhibited. These findings confirm the high efficacy of Chl preparations (even without irradiation), comparable to that of OTC.

On the selective Endo and Sabouraud agars, the growth of microorganisms of the *Enterobacteriaceae* spp. and microscopic fungi was not observed in all groups. This indicates the absence of any additional contamination of the wounds with foreign microbiota.

## 4. Conclusions

The light-independent and photodynamic antimicrobial potential of Chl and Chl-PHB against Gram-positive and Gram-negative microorganisms was shown in the microbiological studies. Irradiation enhanced the toxicity of the preparations by 2 times when exposed to *S. aureus* and *E. coli*, and by 4 times against *B. cereus*. An exposure to doses of preparations equal to 2 × MIC resulted in inhibition of >99% of microbial growth. The highest level of antimicrobial activity was demonstrated by irradiated Chl-PHB. The investigation of the microbial growth inhibition on a solid nutrient medium confirmed the prolonged effect of the polymeric form. Overall, the order of the tested microorganisms’ resistance to Chl was found to be as follows: *E. coli* > *S. aureus* > *B. cereus.* Phase contrast TEM studies discovered the destructive effect of Chl on the microbial cell wall, probably due to the generation of reactive oxygen species. Chl preparations showed significant antimicrobial efficacy comparable to the OTC antibiotic in mice model of infected wound. It was found that irradiation accelerated wound healing and skin regeneration. In summary, based on the data obtained, we conclude that Chl-PHB has potential for use as a bactericidal, wound-healing plaster in the treatment of infected wounds.

## Figures and Tables

**Table 1:** Characteristics of mice experimental groups.

No	Groups	Preparation Dose, µg	Description
1	Control	-	Intact animals
2	Untreated	-	No treatment
3	Chl	360	Chl treatment
4	Chl-PHB		Chl-PHB treatment
5	Chl + light		Chl treatment + irradiation
6	Chl-PHB + light		Chl-PHB treatment + irradiation
7	OTC		OTC antibiotic treatment
8	DMSO	-	DMSO treatment
9	PHB	-	Chl-free PHB treatment

**Table 2:** Mechanical properties of Chl-PHB matrixes.

Content of Chl, %	Strength, N/mm^2^ ± S.D., n = 10	Young’s Modulus, MPa ± S.D., n = 10	Elongation at Break, % ± S.D., n = 10
0	0.24 ± 0.01	0.03 ± 0.01	10.0 ± 0.9
0.5	0.79 ± 0.06	0.50 ± 0.05	1.6 ± 0.6
1.0	0.65 ± 0.08	0.37 ± 0.03	2.4 ± 0.3
1.25	0.46 ± 0.06	0.11 ± 0.02	4.1 ± 0.4
1.50	0.37 ± 0.06	0.40 ± 0.03	2.4 ± 0.4

**Table 3:** Thermo-physical properties of Chl-PHB matrixes, where χ—crystallinity degree, ∆ ±2.5%, ∆H—melting enthalpy, ∆ ±2.5%, Tm—melting temperature, ∆ ±2%, Tc—crystallization temperature, ∆ ±2%.

Content of Chl, %	1 Heating			2 Heating		
	Tm, °C	∆H, J/g	χ, %	Tm, °C	∆H, J/g	χ, %
0	176.4	78.4	52.6	173.5	80.5	54.0
0.5	163.3	44.6	29.9	158.3	38.7	26.0
1.0	163.5	48.4	32.5	159.3	46.8	31.4
1.25	160.2	32.3	21.7	155.7	13.2	8.9
1.50	156.2	25.1	16.8	-	-	-
	**1 cooling**			**2 cooling**		
	**Tc, °C**	**∆H, J/g**	**χ, %**	**Tc, °C**	**∆H, J/g**	**χ, %**
0	73.4	60.6	-	69.1	59.5	-
0.5	102.9	0.5	-	98.5	0.6	-
1.0	102.6	0.5	-	96.0	0.6	-
1.25	103.1	0.5	-	97.5	0.5	-
1.50	109.0	0.4	-	102.0	0.3	-

**Table 4:** Correlation time of TEMPO spin probe in Chl-PHB matrixes, ∆ ±2%.

Content of Chl, %	Correlation Time, s × 10^−10^
0	76.9
0.5	127.1
1.0	109.3
1.25	240.3
1.50	216.8

**Table 5:** MICs values (µg/mL) of Chl and Chl-PHB.

Microorganism	Chl		Chl-PHB	
	No Irradiation	Irradiation *	No Irradiation	Irradiation *
*S. aureus*	12.25 ± 0.30	6.13 ± 0.22	12.15 ± 0.40	5.75 ± 0.20
*E. coli*	36.50 ± 1.40	18.25 ± 0.65	34.5 ± 1.20	17.5 ± 0.75
*B. cereus*	5.93 ± 0.26	1.43 ± 0.07	5.5 ± 0.15	1.25 ± 0.05

* Light dose: 16.2 J/cm^2^, λ = 640 nm. *p*-value is <0.05.

**Table 6:** Preparations-induced reduction in microorganism growth.

Preparations	Reduction in Microorganism Growth, %					
	*S. aureus*		*E. coli*		*B. cereus*	
	MIC	2 × MIC	MIC	2 × MIC	MIC	2 × MIC
Chl	91.93 ± 0.43	99.68 ± 0.09	90.76 ± 0.85	99.28 ± 0.34	93.55 ± 0.52	99.69 ± 0.25
Chl + Irradiation	92.32 ± 0.52	99.72 ± 0.25	91.36 ± 0.93	99.35 ± 0.52	94.81 ± 1.32	99.74 ± 0.18
Chl-PHB	92.67 ± 0.75	99.75 ± 0.12	92.42 ± 1.15	99.47 ± 0.37	95.44 ± 1.21	99.82 ± 0.15
Chl-PHB + Irradiation	94.43 ± 0.64	99.88 ± 0.10	93.68 ± 1.42	99.79 ± 0.20	96.85 ± 0.12	99.89 ± 0.10

## Data Availability

Dataset available on request from the authors. The data are not publicly available due to confidentiality agreements with collaborators or a third party that prevent public release.
